# A Call for a Public Health Agenda for Social Media Research

**DOI:** 10.2196/16661

**Published:** 2019-12-19

**Authors:** Sherry Pagoto, Molly E Waring, Ran Xu

**Affiliations:** 1 UConn Center for mHealth and Social Media University of Connecticut Storrs, CT United States

**Keywords:** social media, online social networks, health information, health communication

## Abstract

Research has revealed both the benefits and harms of social media use, but the public has very little guidance on how best to use social media to maximize the benefits to their health and well-being while minimizing the potential harms. Given that social media is intricately embedded in our lives, and we now have an entire generation of social media natives, the time has come for a public health research agenda to guide not only the public’s use of social media but also the design of social media platforms in ways that improve health and well-being. In this viewpoint we propose such a public health agenda for social media research that is framed around three broad questions: (1) How much social media use is unhealthy and what individual and contextual factors shape that relationship; (2) What are ways social media can be used to improve physical and mental well-being; and (3) How does health (mis)information spread, how does it shape attitudes, beliefs and behavior, and what policies or public health strategies are effective in disseminating legitimate health information while curbing the spread of health misinformation? We also discuss four key challenges that impede progress on this research agenda: negative sentiment about social media among the public and scientific community, a poorly regulated research landscape, poor access to social media data, and the lack of a cohesive academic field. Social media has revolutionized modern communication in ways that bring us closer to a global society, but we currently stand at an inflection point. A public health agenda for social media research will serve as a compass to guide us toward social media becoming a powerful tool for the public good.

## Introduction

Recent leaks of Facebook Chief Executive Officer (CEO) Mark Zuckerberg warning employees about the implications for Facebook if a top Democratic candidate became president resulted in yet another wave of negative press for the company [1], a continuing backlash initially provoked by a 2014 Facebook research study that manipulated user newsfeeds [2,3]. The hashtag “#deleteFacebook” often goes viral following these incidents, as angry users publicly vow to disconnect their accounts [4]. Despite the backlash, in 2019, the Pew Research Center reported no decline in Facebook users over this period [5]. Although many people seemingly have serious concerns about Facebook and other social media platforms, social media bring a particular value to people’s lives that make us reluctant to disconnect. To the extent that people enjoy and even depend on the community and resources that social media bring to their lives, disconnecting may be felt like more of a loss than a gain. We know surprisingly little about the value that social media bring to people’s lives.

Social media is a burgeoning area of study in the field of public health; however, much research covered in the popular media has focused on its harms [6-9]. Persistent negative headlines drive a narrative about social media that likely deteriorates public sentiment. To be sure, enough research has been conducted to know that some uses of social media are indeed harmful, but the public has very little guidance on how to modify their use to maximize the benefits to health and well-being while minimizing the potential harms. Given that social media are intricately embedded into our lives and we now have an entire generation of social media natives, the time has come to create a public health research agenda to guide the use and design of social media in ways that improve health and well-being. In this viewpoint, we propose such a public health agenda for social media research that is framed around three broad questions (Textbox 1):

- How much social media use is unhealthy, and what individual and contextual factors shape that relationship?
- What are the ways social media can be used to improve physical and mental well-being?
- How does health (mis)information spread, how does it shape attitudes, beliefs, and behavior, and what policies or public health strategies are effective in disseminating legitimate health information while curbing the spread of health misinformation?

**A public health agenda for social media research: three areas of inquiry and specific research questions.**

1. How much social media use is unhealthy, and what individual and contextual factors shape that relationship?
  - Which social media activities lead to positive versus adverse health outcomes?
  - What are the individual and contextual determinants of social media use leading to positive versus adverse health outcomes?
  - What factors predict change in time spent in health-promoting versus harm-promoting social media activities?
  - What is the impact of change in time spent in health-promoting versus harm-promoting social media activities on health outcomes?
  - How can we develop more granular measures that capture users’ time spent engaging in specific social media activities?
2. What are the ways social media can be used to improve physical and mental well-being?
  - What is the impact of health-related initiatives led by social media platforms and public health organizations?
  - What strategies are most effective in generating meaningful patient engagement (ie, the type of engagement that leads to healthy changes in knowledge, attitudes, and behavior) in online communities?
  - What factors influence whether health-related advocacy on social media is efficacious at shifting public attitudes or influencing the development and implementation of public health policy?
  - How can commercial platforms and public health organizations harness effective social media interventions in scalable ways that can impact public health?
3. How does health (mis)information spread, how does it shape attitudes, beliefs, and behavior, and what policies or public health strategies are effective in disseminating legitimate health information while curbing the spread of health misinformation?
  - What are the characteristics of health misinformation and disinformation, of their messengers, and the means they are using to do so?
  - How do we characterize the social bot and troll ecosystem around health-related messaging and how it evolves?
  - What are the characteristics of accurate health information messages that spread on social media, and how do these spread?
  - Who are the audiences of health-related organizations’ social media feeds, and to what extent does their messaging reach people who are at the highest risk for the target health condition?
  - What are the characteristics of social media spaces in which health misinformation and disinformation are rare, and how has that occurred?
  - How does legitimate health information shared on social media impact attitudes, beliefs, and behavior, and how does this compare to the impact of health misinformation and disinformation?
  - How do the characteristics of both the messenger and the message recipient affect message receptivity?
  - What are effective health literacy education programs that can decrease susceptibility to health misinformation and disinformation?
  - How do we develop effective counter-messaging to health misinformation and disinformation that adapts to the evolving challenges of the modern social media landscape?

## Public Health Agenda for Social Media Research

### How Much Social Media Use Is Unhealthy and What Individual and Contextual Factors Shape That Relationship?

Although studies demonstrating an association between greater social media use and poor health outcomes (eg, depression, loneliness, poor body image) [10-13] are plentiful and make for popular headlines, the literature on social media use and well-being is quite mixed [14]. Indeed, a recent meta-analysis of 31 studies found that higher social media use was associated with more significant online and offline social support [15]. Nonlinear relationships have also been observed such that both heavy and no social media usage have predicted worse mental health. Increasingly studies are pointing to moderating variables that influence the relationship between social media use and health-related outcomes [16,17]. At this point, studies that report simple associations between time spent broadly on social media and a specific health outcome do little to advance our knowledge about the impact of social media use on health [18]. More research on specific types of social media activities, the individual and contextual determinants of these activities, and how they lead to positive versus adverse health outcomes is now very much needed [19]. Further, longitudinal studies that identify factors that predict change in time spent in health-promoting versus harm-promoting social media activities and the impact of changes in time spent in these activities on health outcomes would create knowledge that could inform public health guidelines [19].

Given how little we know about how to define the boundaries of problematic social media use, it is premature to devise solutions to curb it. Nevertheless, solutions are being proposed, most of which involve calls to limit social media use [20]. Notably, Senator Josh Hawley proposed the Social Media Addiction Reduction Technology (SMART) Act [21]. If it is passed, it will ban social media platform features assumed to promote compulsive use, such as infinite scrolling and autoplay, while requiring platforms to include features that implement time limits. However, little research has explored whether infinite scrolling and autoplay features negatively impact users.

Further, encouraging time limits on social media usage overlooks that not all users have negative experiences on social media, and not all social media uses are inherently harmful. Policy and public health recommendations that focus on quantity instead of quality essentially equate a patient spending 6 hours a week engaging with her cancer Facebook group to help her cope with treatment to a cyberbully spending the same amount of time harassing classmates on Snapchat. Evidence-based guidelines are needed to prevent real harm that can result from discouraging or shaming social media use in people whose well-being is enriched by, or even dependent upon, their social media activities.

New self-report measures are emerging that allow researchers to assess the degree to which one’s social media experiences are negative or positive [22,23]. Tools like this could be useful as we attempt to understand which specific uses are associated with negative and positive experiences and health outcomes. More objective and granular measures that capture time spent engaging in specific social media activities, such as engaging with a particular Facebook feed or group, are also needed to advance the research agenda. Currently, no such tools exist [24]. While self-reported measures are easy to administer, self-reported estimates of time spent in activities that are brief, unplanned, and unstructured (such as typical social media use [25]) are less accurate compared to estimates of time spent in more structured and prolonged activities, such as time spent working or commuting [24]. While there are software programs and mobile tools that track time spent using Facebook via a mobile app or web browser on a computer (eg, Screen Time [iPhone], Stay Free app [Android]) [26], these tools do not capture use across multiple devices. Software-tracked time also does not capture how much time the participant spent engaging in different activities. Until new tools to objectively measure specific social media use becomes available, ecological momentary assessment could be leveraged to track time spent engaging in specific social media uses, along with factors such as positive and negative affect, body dissatisfaction, sense of belonging, and loneliness, to provide insights into the relationship between specific uses and these outcomes. This approach could also be leveraged in interventions in which users self-monitor their social media activities and these factors, receive feedback about these relationships and set goals that maximize positive uses while minimizing negative uses.

### What Are Ways Social Media Can Be Used to Improve Physical and Mental Well-Being?

Some commercial social media platforms are investing in health-related tools that have promising public health implications. For example, Facebook partnered with the American Blood Centers and the American Red Cross to produce a blood donation tool that alerts users to blood supply shortages in their area and notifies them of locations where they can donate [27]. Also, Facebook, Instagram, Pinterest, and Twitter all provide suicide hotline information to users who use suicide-related search terms. Pinterest removed antivaccine content on its platform and adopted a policy where vaccine-related searches produce health information from leading public health organizations [28]. Related, Facebook and Instagram reduced the ranking of groups and pages that spread misinformation about vaccines, rejected ads for vaccine-related misinformation, and provided pop-ups for searchers using vaccine-related search terms [29]. YouTube has pulled ads from videos containing antivaccine content to prevent sites from monetizing this content [30]. That commercial social media platforms are taking actions to improve public health is a promising trend. Very little research has explored the impact of these initiatives, most of which are very new. Facebook offers research awards on specific topics [31], but the public health community should attempt to shape their agenda. As is beginning to occur in the social sciences [32], partnerships between commercial platforms and public health scientists are needed to research the impact of these initiatives and to inform the development of new public health initiatives.

Users are also discovering unique ways to leverage social media to improve their health. Online patient communities have proliferated on social media as a way for patients to connect with one another on specific health topics. “Peer-to-peer healthcare,” a term coined in 2011 by Susannah Fox at the Pew Research Center, refers to patients using social media to connect with other patients about their health [33]. Online patient communities have organically formed on nearly all social media platforms. They are often patient-initiated, patient-moderated, and open forum, meaning conversation threads can be initiated by members as they wish. Research has begun to examine online patient communities, and the benefits members gain from participation. For example, a review of 47 studies of online diabetes communities on multiple platforms found evidence for positive clinical, behavioral, psychosocial, and community outcomes, and very few negative outcomes [34]. A survey study of a breast cancer community on Twitter found that 80.9% of respondents reported that participation increased their breast cancer knowledge, 66% learned of clinical trial opportunities, 31% learned information that led them to seek a second opinion, 71.9% reported being inspired to increase their advocacy efforts, and 67% of those with high anxiety about their diagnosis reported declines in anxiety [35].

Online patient communities may even impact health care costs. A longitudinal study of new enrollees of HealthUnlocked, a collection of online patient communities in the United Kingdom, revealed declines in emergency room visits [36]. Much more research is needed to fully understand the range of benefits people experience in these communities and how to amplify beneficial experiences. This research faces logistical and ethical challenges because many online communities are private, and the sheer size of them makes obtaining informed consent from all members difficult and unlikely. Further, the presence of researchers may not be desirable in spaces created by patients for patients [37]. For this reason, a community-based participatory research model, adapted for online communities, may be needed to create partnerships. Nevertheless, researchers must establish the value proposition for online community leaders and members to engage with them.

Online patient communities are used not only for peer-to-peer support but also for advocacy efforts. Donna Helm Regen, moderator of the Pull the Plug on Tanning Beds Facebook page [38], lost her daughter, a tanning bed user, to melanoma before the dangers of tanning beds were well-publicized. She uses her Facebook page to inform the public about the harms of tanning beds and advocate for tanning bed legislation, often in partnership with nonprofit organizations and other advocacy groups with whom she has connected with on social media. Her efforts afforded her opportunities to make a national public service announcement [39] and to testify at state legislative hearings supporting tanning legislation. Social media give grassroots health advocacy efforts a platform to shore up support for their cause. This is another example of how encouraging arbitrary social media use limits may inadvertently squelch important public health efforts. People have discovered ways to use social media to impact their health, their community’s health, and public health, much of which has gone entirely unstudied. Research on what makes these efforts work and how to enhance their efficacy is needed to strengthen their impact on changing individual behavior, shifting public attitudes, and influencing public health policy.

Patients are not the only ones creating online health communities. Researchers have begun to create online patient communities to implement and evaluate the efficacy of social media-delivered behavioral interventions and public health campaigns [40]. Typically, investigators will start a private online forum on a commercial social media platform (eg, private Facebook group), recruit members of a target population to join the group, host a feed that delivers behavioral intervention or public health messaging for some time, and then measure outcomes. Often these programs employ a health care professional or health coach as a moderator. Systematic reviews of studies evaluating such interventions for weight management [41], smoking [42], HIV testing [43], and cancer prevention [44] reveal promising outcomes. These online communities provide the benefit of both peer-to-peer support and evidence-based health information. However, this model has drawbacks. Researcher-derived communities are smaller than online patient communities in the real world, which raises concerns about scalability. They are also time-limited, which necessitates aggressive engagement strategies to strengthen ties between members of the community.

Further, we know of no examples of evidence-based social media interventions having been implemented in a real-world setting. Many nonprofit public health organizations have online patient communities, but do not provide health promotion interventions within those communities, which presents an opportunity for partnerships with intervention researchers. The closest example is the Truth Initiative’s “Become an Ex” community for smokers [45]. Their platform provides evidence-based online resources for smoking cessation that community members can use to assist their attempts to quit. Much research is needed to examine how to generate meaningful patient engagement in online communities and how to scale these models for implementation at the community or population level [46].

The promising data emerging about the health benefits of online patient communities support the notion that online social interactions that occur in tighter-knit communities created based on shared life experiences may have the most potential to impact health and well-being. A movement towards a tighter community-focused model of social media appears to be afoot at Facebook and Instagram. In March 2019, CEO Mark Zuckerberg announced [47]:

Over the last 15 years, Facebook and Instagram have helped people connect with friends, communities, and interests in the digital equivalent of a town square. But people increasingly also want to connect privately in the digital equivalent of the living room. As I think about the future of the internet, I believe a privacy-focused communications platform will become even more important than today's open platforms. Privacy gives people the freedom to be themselves and connect more naturally, which is why we build social networks.

To the extent that social media use shifts toward private forums built around shared life experiences, the quality of interactions and impact on well-being is likely to improve, which may, by design, diminish negative experiences. Research should inform the type of shift in platform features, tools, and design needed to shape users’ experiences in this way.

### How Does Health (Mis)information Spread, how Does It Shape Attitudes, Beliefs and Behavior, and What Policies or Public Health Strategies Are Effective in Disseminating Legitimate Health Information While Curbing the Spread of Health Misinformation?

#### Overview

Long before the term “fake news” was coined, concerns were expressed regarding the veracity of health claims online and elsewhere [48]. Bogus health claims come in two forms: misinformation and disinformation. Health misinformation refers to information that is false and spread by someone who believes it to be true, whereas health disinformation refers to information that is false and spread by someone aware it is false and is intent on misleading people [49]. The volume of both on social media has grown into a public health epidemic [50]. While research is nascent on strategies to combat this problem, a multi-prong approach is needed, including curbing its spread, inoculating people against false messages, and developing effective counter-messaging. Tackling this complex and urgent public health problem will require an interdisciplinary research agenda.

#### Curbing the Spread of Health Misinformation

To curb the spread of health misinformation and disinformation on social media, we must first have the means to identify three things: (1) false messaging; (2) messengers who spread them and the reasons why (ie, misinformation or disinformation); and (3) the means they are using to do so. While studies of health misinformation on social media have covered a variety of health topics, including infectious disease (eg, vaccines [51], antibiotics [52], cancer [53,54], electronic cigarettes [55], eating disorders [56,57], and nutrition [58,59]), a recent systematic review of 57 health misinformation studies revealed this area of work is dominated by infectious disease studies [60]. This review reported that the most common purveyors of health misinformation appear to be from people with no institutional affiliations.

Studies of “social bot” activity reveal that these accounts flood the conversation on particular health topics [61,62]. Social bots are social media accounts designed to appear to be manned by a human, but they are automated to put forth or respond to content in specific ways [63]. Not all social bot accounts have nefarious intentions, with some instead serving helpful purposes, such as content aggregation [63] or gamifying health challenges [40]. Trolls are accounts operated by humans who are intentionally disruptive towards others and may or may not be compensated for doing so [64]. One study of antivaccination content spread by “social bots” and “trolls” found that these accounts are pervasive and designed to create false equivalency and sow discord about vaccines [61]. Social bot and troll accounts rapidly proliferate, which gives them extraordinary power to flood social media with messaging in ways that convey the false sense that a certain message is popular or well-accepted [63]. The use of social bots to conduct misinformation campaigns may be one reason health misinformation (and disinformation) is so much more plentiful on social media than legitimate health information [60].

Commercial platforms have developed algorithms to identify the nefarious activities of bots and trolls, to reduce these activities and delete their accounts [65]; however, new accounts designed to circumvent the algorithms eventually emerge, making for a seemingly never-ending cat and mouse game [63]. Troll accounts are exceptionally resistant to algorithm detection, being manned by real people who may be paid to do so [64]. Surveillance studies are needed to understand the bot and troll ecosystem around health-related messaging and how it is evolving. Further, while social bots have been used for ill-intentions, research should examine how they may be used for good. For example, as part of an anti-bullying public service announcement, Monica Lewinsky released the “Goodness Bot,” a Twitter account that, when mentioned in reply to a bullying tweet, responds with a positive message [66].

Research is also needed to improve our understanding of how legitimate health information is produced and spreads on social media. When it comes to health misinformation, a strong defense may require a strong offense. Many public health departments and nonprofit organizations use social media to disseminate health messaging. A study of state public health departments revealed that most have a social media presence [67]. Another study specifically focusing on state public health departments’ use of video-based messaging found that 43/51 departments have YouTube channels with a total of 6302 subscribers, 3957 videos, and 12,151,720 views [68]. Most large, health-related, nonprofit organizations also have a social media presence. A study of 24 of the largest nonprofit skin cancer organizations’ Facebook feeds found that the organizations had a collective total of 225,113 followers and in one year produced 824 posts that received 92,004 likes, 4148 comments, and 82,791 shares [69]. Interestingly, the message types that received the most shares were fear appeals and myth busters. While these organizations seem to be producing impressive reach, it is unclear how these numbers compare to the reach of health misinformation on the same topics. For example, a recent study of 1000 tweets using the words “tanning bed” and “tanning salon” revealed that most were made by tanners expressing positive sentiment about tanning, with only 4.3% of the tweets containing health warnings [70]. This suggests that skin cancer organizations messaging may not be penetrating social media spaces where people at risk are discussing their habits. More research is needed to understand the audiences of health-related organizations’ social media feeds, the extent to which their messaging reaches people who are at highest risk for the target health condition, and the impact of their messaging on attitudes, beliefs, and behavior. Research is also needed to inform health organizations’ social media–messaging content strategy.

While health misinformation and disinformation may be abundant in some spaces on social media, we do not know much about the social media spaces that have very little and how that has occurred. For example, a review of online diabetes communities revealed very low rates of health misinformation [34]. This suggests that health misinformation is not a given in online spaces where patients connect but instead may have the tendency to flourish under specific circumstances. Some communities may self-police health misinformation, but how effective they are depends on their ability to identify it. Moderators of online patient communities, who are essentially a self-appointed army of volunteer community health workers, have very little training and guidance on how to identify and stem health misinformation, but many are likely motivated to do so. Public health researchers can inform the work of moderators, but we see few examples so far of such partnerships.

#### Health Misinformation Inoculation

Inoculating the public against health misinformation (and disinformation) requires an understanding of whether and how they influence attitudes, beliefs, and behavior, and who is susceptible to being duly influenced. The mere existence of health misinformation on certain topics does not necessarily mean it is changing people’s attitudes and behavior, and the same goes for legitimate health information. However, exposure to health misinformation may strengthen preexisting misinformed beliefs. Some evidence suggests that users seek out messaging and online communities that confirm their preexisting beliefs [51]. That said, certain message characteristics may influence message effectiveness. Recent measles outbreaks strongly suggest that antivaccination messages have had an impact on beliefs and behavior [71,72]. These messages often stoke fear or leverage conspiracy theories, which we know to be particularly effective message strategies [73]. Message effectiveness also depends on the characteristics of the messenger and the recipient. Transportation theory suggests that narrative messages are particularly powerful at affecting beliefs, attitudes, and behavior to the extent that people identify with the person sharing their story [74]. Research on the extent to which misinformed narratives versus health misinformation conveyed in other forms (eg, links) is more powerful for affecting message recipients is needed. Chou and colleagues at the National Cancer Institute have called for research into how characteristics of both the messenger and the message recipient affect message receptivity [75].

Scientists, health care professionals, and medical journal editors have been called on to be messengers of evidence-based health information [76]; however, they have little evidence-based guidance on how to do so. One study of the impact of scientist demeanor on message credibility revealed that scientists exhibiting hostility in a debate were found to be less credible and trustworthy [77]. Knowledge about the complex relationships between message, messenger, and recipient is necessary to inform strategies that inoculate people against health misinformation. Finally, health literacy training is an inoculation strategy that may be useful in primary to post-secondary educational settings [75,78]. Low health literacy has been shown to affect how people evaluate health information they encounter online [79]. Given the volume of health information on social media (and the internet in general), children and adults alike require skills for sorting, vetting, and processing this information, as well as an understanding of how cognitive biases affect information processing. Research on health literacy education that effectively decreases susceptibility to online health misinformation is much needed.

#### Developing Effective Counter-Messaging

Some, albeit limited, research has focused on developing effective counter-messaging. For example, one study found that platform-based warnings such as “this tweet may contain misinformation” decreased the likelihood of the post being shared [80]. Similarly, another study revealed that misinformation correction by platforms and peers reduced misperceptions [81]. A significant challenge to developing effective counter-messaging is that some groups may be especially resistant to it, such as those who have already strongly embraced misinformation. Individual characteristics, such as the sunk cost investment in the misinformation (eg, time and resources spent disseminating it), the negative consequences of changing positions (eg, embarrassment, loss of community/social capital, shame about harm that may have occurred from action or inaction relating to the misinformation), and literacy, likely affect resistance [60]. Researchers focused on developing counter-messaging campaigns should leverage the expertise of human computing interaction and social media marketing experts who can help guide design and dissemination strategies that are best matched to the social media platform. A great need exists to update traditional health communication theories and strategies that were not informed by the unique form of communication afforded by social media and the ever-evolving challenges of the modern social media landscape.

### Challenges to a Public Health Agenda for Social Media Research

#### Overview

Four key challenges constrain progress on a public health agenda for social media research: (1) negative sentiment about social media and villainization of social media companies among the public and scientific community; (2) a poorly regulated research landscape; (3) inadequate access to social media data; and (4) lack of a cohesive academic field.

#### Negative Sentiment

Negative public sentiment about social media may be part of society’s general tendency to push back on new technology, a phenomenon that has a long history [82]. To be sure, scandals involving how social media companies handle privacy and data access have amplified the public response. The consequences of a persistently negative narrative are that it makes it difficult to shore up support for healthy solutions, which may result in an abrupt dismissal of signs of progress. To the extent that the academic community adopts a defeatist view about social media, we may squander our opportunity to shape the social media landscape in ways that improve public health.

#### Poorly Regulated Research Landscape

Research involving social media platforms is emerging rapidly in a poorly regulated landscape [83]. Federal guidelines for the ethical conduct of research do not specifically address the unique ethical challenges of working with social media data [84]. As such, scientists and institutional review boards (IRBs) have little guidance for addressing the ethical, legal, and social implications of social media data use. A qualitative study of university IRB members revealed concerns that investigators do not adequately describe potential risks or have clear plans to minimize risks in IRB applications, IRB members often do not have the knowledge to review such protocols adequately, and that IRBs are having difficulty keeping pace with rapidly changing technologies used in research [85].

As discussed in two recent reviews [86,87], privacy, confidentiality, and informed consent are key ethical issues in social media research [88-90], though specific ethical considerations differ across study types (eg, surveillance, surveys or interviews, interventions) [91]. While not all research involving social media data meet the federal definition of human subjects research, and thus do not require informed consent, researchers still need to be aware that users may not realize that their public social media content can be used in research studies and may prefer that it not be used this way [88]. Even though use agreements address the potential for public social media content to be used in research and otherwise, the majority of users accept lengthy, difficult-to-read, use agreements without reading or understanding how their data can be accessed and used [92]. Standard rules and protocols to guide social media data use, as well as data sharing between industry and academia, are needed and must be rigorous in terms of privacy and data security. One collaborative working towards this goal is ReCODE Health [93], an academic group that has created a community for resource-sharing (eg, sample IRB documents) and education about the protection of human subjects [94]. For studies that require informed consent, we encourage researchers to add detailed information about the procedures for the collection, storage, and analysis of social media data [89]. For studies involving publicly posted content, we suggest researchers refrain from including exact quotes, which can be traced back to users in ways that could have the potential for harm [88-90].

#### Data Access

Access to much social media data is limited, including both public data and data that users give researchers consent to access. Following the Cambridge Analytica scandal, Facebook and Instagram changed their application program interfaces (APIs) to restrict data access by third parties [95]. While restricting data access seems to be a plausible solution, it also limits how data can be used for the public good. Furthermore, frequent changes to data access rules require researchers to face the exorbitant costs of acquiring data from third party applications that are agile to API changes but typically geared for commercial use. Such tools also add another layer of risk for privacy breach. When data access changes during a research study, it can compromise the ability to answer key research questions, forcing researchers to redesign studies midstream or compromise research quality.

Facebook’s partnership with Social Science One, an organization designed to facilitate academic-industry partnerships “to advance the goals of social science in understanding and solving society’s great challenges,” [96] is a promising example of how such partnerships can work [97]. Social Science One has developed an industry-academic partnership model that is designed to navigate barriers to data access, such as consumer privacy, trade secrets, and proprietary content, within a structure that is committed to securing the trust of the academic community and the general public [32]. Facebook partnered with Social Science One to allow scientists to access Facebook data to study how social media influences democracy. Progress has moved slowly [98], and results of the partnership remain to be seen, but this effort is the first of its kind to tackle the challenges of academic-industry partnerships involving social media data.

The barriers to access to social data by academic researchers is deeply troubling. Academic researchers have limited ability to conduct rigorous research that could change the way people use social media for the better. Technology and marketing companies have a greater ability to afford and access social media data, face fewer regulations, and have less oversight, particularly as it relates to protecting users. Companies also monetize this data in ways that are not transparent to the public [78]. More research is critically needed to develop new business models and technologies to protect user privacy while facilitating collaboration and data sharing between industry and academia [99].

#### Lack of a Cohesive Academic Field

Social media research has flourished in recent years, but a cohesive field has not yet emerged to bring this scientific community together. A cocitation analysis of 121 journals that have been cited at least five times in studies of health misinformation on social media revealed four disciplinary clusters: social psychology/communication, general science/medicine, infectious disease/vaccine and public health, and medical internet and biomedical science [60]. While the breadth of fields involved in this research is encouraging, several important fields are missing, including human computing interface, engineering, data science, and computer science. The researchers also found limited cross-citations between the psychology/communication cluster and the general science and medicine cluster, suggesting that researchers in different silos may not be exposed to each other’s work. The lack of a scientific field not only disperses this research across disparate journals but also across myriad scientific conferences, which provides few opportunities for scientists doing this work to build collaborations. Scientific networking opportunities are needed, as well as more transdisciplinary training programs. For example, public health training programs that offer coursework in analytic approaches, such as machine learning, natural language processing, and social network analyses, would better equip public health scientists to do this work; just as ethics, social science, health communications, and health policy coursework could better equip computer scientists, engineers, and data scientists to do this work.

## Discussion

Social media has revolutionized modern communication in ways that bring us closer to a global society than ever before. How social media has been used thus far reflects the full range of human proclivities. However, we have the power to shape its course. The field of public health is an obvious leader in the charge to inform the use and design of social media to benefit public good. We put forth a public health agenda for social media research, not to be exhaustive, but to start a dialogue about the need for such an agenda. Other areas of research to be further explored include public health surveillance with social media data, social media marketing of healthy and unhealthy products, behavioral pattern analysis, social-behavioral biometrics, and pharmacoepidemiology, to name a few. Federal funding agencies can take the lead in shaping the course of this work by prioritizing it in their strategic plan and among their funding opportunities. The vast range of uses of social media data speak to the need to finally convene an interdisciplinary scientific field devoted to public health social media research.

## Abbreviations

API: application program interface
CEO: Chief Executive Officer
IRB: institutional review board
SMART: Social Media Addiction Reduction Technology
